# 

**Published:** 1871-05

**Authors:** 


### CONTENTS:

ORIGINAL COMMUNICATIONS:

ARTICLE I. Miscellaneous Practice. By F. B. Norcom, M.D. ............ 257

ART. II. On a few Instruments for the Practical Treatment of Uterine Disease. By Philip Adolphus, M.D. Third Paper ................ 267

ART. III. Correlation of Physical and Vital Force; Clinically Illustrated. By Z. C. McElroy, M.D.. 276

ART. IV. Cases from the Note Book of Service in the Medical Department of the U. S. Army. By Chas. M. Clark, M.D., late Surgeon 39th Ill. Vols ....................... 288

ART. V. Emboli. By Walter Hay, M.D ............................... 291

ART. VI. Case of Punctured Wound of the Brain—Recovery. By C. T. Fenn, M.D ....................... 294

EPISTOLARY ....................... .... 295

EDITOR'S BOOK TABLE ................. 302

EDITORIAL ........................ .. 306

OBITUARY ............................ 310

NOTICE ......... ...................... 313

LOOT ............................ ..... 314

